# New Advances in Strengthening of Structural Timber

**DOI:** 10.3390/ma17112545

**Published:** 2024-05-24

**Authors:** Paweł Grzegorz Kossakowski

**Affiliations:** Faculty of Civil Engineering and Architecture, Kielce University of Technology, 25-314 Kielce, Poland; kossak@tu.kielce.pl

As one of the oldest building materials, wood is still widely used today. This is largely due to its relatively high strength and low weight. Wood’s excellent environmental credentials are not insignificant either. However, timber structures often need to be repaired and strengthened. This is mainly due to wood’s susceptibility to various factors that cause its mechanical parameters to deteriorate, which is particularly noticeable in very old, historic buildings. This has led to a constant search for new and better ways to repair and strengthen wooden structures.

Increasing the load-bearing capacity and stiffness of engineered timber is often achieved by reinforcing both new and existing members. Bars, sheets (unidirectional, bidirectional, or multidirectional), laminates, or profiles are typically used as these reinforcements. Traditional reinforcing materials, such as steel or other metals such as aluminium, are often used. However, due to their excellent mechanical properties and relatively low weight, composites are now increasingly being used. Fibre-reinforced composites (FRC) are the basis here. Aramid, glass, carbon, or basalt fibres embedded in a resin matrix are becoming increasingly popular.

To improve performance, traditional passive reinforcement can be replaced by active reinforcement, which has the significant advantage of introducing pre-camber into the component or using a pre-stressing system. Although a great deal of research has been carried out on the strengthening of timber and many methods and technologies have been developed, there is still a lot of work to be done. This includes developing new wood-based materials, innovative structural timber components, and new reinforcing materials and strengthening technologies.

This Special Issue explores recent advances in the field of strengthening structural timber. It includes theoretical, experimental, numerical, and practical research on the analysis, description, and optimisation of innovative methods and technologies that have been developed and implemented for the strengthening of various types of wood-based structural members.

For decades, timber structures and members have been primarily reinforced with metallic materials, most commonly steel members [1,2]. Today, these technologies can be considered traditional, as they are proven and effective in ensuring reliable building operations [3]. Typical solutions include timber-steel composite structures, where steel sections act as reinforcing elements. A more modern approach is to use steel rods or flat bars bonded in slots cut into the timber, similar to classical reinforced concrete [1,2].

An interesting avenue to explore in this area is research into wood-steel hybrid elements for use as structural components. The first articles on the topic appeared in the eighties [4]. Since that time, hybrid systems have not developed into significant alternatives to other approaches. A review of research in the field of timber-steel hybrids for beams is given in [5]. Long-term tests of hybrid bending beams have shown that bending stiffness and load-bearing capacity can be significantly increased (3–4 times) compared to pure timber beams. The importance of material joining technology has been confirmed [6]. In this context, bonding is particularly important. Recent work has also focused on hybrid columns under static and dynamic loading, e.g., [7,8].

It is very important to note that hybrid elements as well as all-wood constructions can be alternatives to traditional systems and material solutions that meet the requirements of green building. In this context, the current sustainability policies and requirements for new investments in some countries are interesting. In the German state of Baden-Württemberg, for example, every new public building must be designed and built using timber or a timber-hybrid structure (Holzbauoffensive BW). To meet this challenge, a recently completed research project at the Karlsruhe Institute of Technology aimed to develop and systematically test hybrid bending beams using an advantageous combination of steel and timber materials. The article [9] presents the research carried out and the results obtained on hybrid elements—steel profiles integrated into timber cross-sections in a shear-resistant manner by means of adhesive bonding. Experimental, numerical, and analytical studies were carried out on different steel and timber cross-sections and different construction materials (i.e., GL24h, LVL48p, LVL80p, S355, and S420). The potential of this new hybrid design is illustrated by the results of large-scale, four-point bending tests. The bending stiffness can be increased by up to 250% and the load-bearing capacity by up to 120% compared to a glued laminated timber element of the same dimensions, depending on the geometry and material combination tested.

Today, solid wood is reinforced with various types of fibre composites, most commonly carbon, glass, aramid, or basalt fibres. Composite laminates, sheets, and rods are used to reinforce structural elements. In this respect, research into the use of composites is innovative, as they have previously been used to reinforce materials other than wood, notably reinforced concrete. The research presented in this issue [10] is part of this trend.

This paper discusses the performance of bent solid wood beams reinforced with a FRCM-PBO (fibre-reinforced cementitious matrix-p-phenylene benzobisoxazole) composite. The FRCM system is designed to strengthen concrete, reinforced concrete, and masonry structures. Tests on bending beams reinforced with PBO-FRCM have shown that this system provides effective reinforcement, but the increase in load capacity is less than that of FRP reinforcement [11,12,13]. However, it has not yet been tested on timber structures. In the study [10], ten pine beams, each with a cross-section dimension of 80 mm × 80 mm and a length of 1600 mm, were tested. Five of the beams were unreinforced and served as control specimens, while the other five were reinforced with the FRCM-PBO composite. A layer of mineral resin and quartz sand was applied to improve adhesion between the FRCM-PBO composite and the wooden beam. The results showed a significant increase in load-bearing capacity of 141.5%, maximum bending stress of 118.9%, Young’s modulus of 18.3%, time to failure of 106.6%, and deflection of 115.6% compared to the control samples. The unusual method of wood reinforcement presented in the article can be considered innovative, not only because of its significant load-bearing capacity but also because of its simplicity of application.

Although the technology to produce composite materials has been developed and is widely used, new solutions are still being sought. One of the more innovative approaches is the use of 3D printing. The materials produced in this way require extensive testing before they can be used, mainly for strength but also for durability and resistance to various factors. In combination with polymers and other materials, there is considerable interest in applying 3D printing to wood-based materials [14]. It contributes to increased sustainability. In addition, 3D printing offers the opportunity to create innovative structural shapes [15]. An interesting approach is to combine 3D printing with wood-based materials [16].

Research has been carried out into the applicability of 3D-printed composites for wood reinforcement, the results of which are presented in the paper [17]. The aim of this study was to investigate the differences in the mechanical properties of solid structural timber and timber reinforced by three different methods. Wood elements reinforced with carbon fibre-reinforced polymer (CFRP), 3D-printed polycarbonate (3DPC) laminates, and 3D-printed polycarbonate with carbon fibre (3DPCCF) laminates were subjected to a three-point bending test. The CFRP samples showed an 8% bending strength advantage over the 3DPCCF samples and a 19% advantage over the 3DPC samples. On the other hand, when theoretical manufacturing costs are considered, the performance of 3DPCCF is almost three times higher than that of CFRP and 3DPC. Furthermore, 3D materials allow more complex reinforcement shapes than this paper describes.

Although solid wood is still the basic material for many building structures, glued laminated timber (commonly known as glulam) is increasingly being used. Due to its excellent strength properties at relatively low density and its wide range of geometric possibilities, as well as its durability and corrosion resistance, it is widely used in long-span buildings and special functions. In addition to improving glulam production technology, work is being done on hybrid glulam composites to further improve their mechanical properties.

Laminated veneer lumber (LVL) is a type of glued laminated timber that is widely used and has excellent performance characteristics. The reinforcement of LVL with composite materials is carried out using the same materials as those used for solid wood; laminates and sheets are the most commonly used. Engineering wood products based on poplar veneers and composite materials have great potential for structural use [18]. Three articles in this Special Issue deal with the reinforcement of LVL wood [19,20,21].

The first study [19] presents the results of experimental tests on the bending strength and modulus of elasticity of unreinforced and reinforced seven-layer LVL poplar veneer panels. The aim of the research is to investigate the effect of woven carbon fibres on improving the bending properties and stiffness of LVL bending in the sheet plane and to evaluate the effect of adhesives on the flexural properties of composite products. This is to assess the potential use of the material as a structural element. Tests were carried out on small samples with three different reinforcement configurations and a control sample using either epoxy or melamine urea formaldehyde (MUF) adhesive. The study found that using carbon fibre-reinforced polymer (CFRP) with epoxy adhesive significantly improved bending strength and bending modulus of elasticity. Average improvements in bending strength for the reinforced specimens ranged from 32.9% to 38.7%, while improvements in bending modulus of elasticity ranged from 50.7% to 54.7% compared to the control specimens. In addition, reinforced samples showed different behaviour in terms of elasticity and plasticity during testing. However, melamine-urea formaldehyde adhesive was not effective in forming a composite product with veneer and carbon fabric. The research demonstrates the potential of using poplar veneer in the design of LVL structural elements when using epoxy adhesives.

When testing wooden components, it is very important to obtain reasonably reliable results, which is sometimes difficult due to the high anisotropy of wood, especially solid wood. In the case of glulam, this effect is, of course, reduced by the choice of component material and a high production regime. In this respect, 1:1 tests on technical or natural-scale components are very valuable.

Such research is described in the article [20], where the results of tests on LVL elements reinforced with composite sheets are presented. The study investigates the effect of reinforcing full-size LVL beams with carbon fibre-reinforced polymer (CFRP) sheets. Unreinforced and reinforced LVL beams with nominal dimensions of 45 mm × 200 mm × 3400 mm were investigated. The U-type reinforcement method was applied in three configurations of varying thickness and side surface coverage. An epoxy resin adhesive bonded the components together. A four-point static bending test was carried out in accordance with European standards. The results showed that the effectiveness of the reinforcement increased with the level of side surface coverage and the level of reinforcement. The average increase in bending strength was between 42% and 58% for the configurations analysed. In turn, the average bending stiffness increased from 15% to 43%. Reinforcement changed the failure mode from brittle fractures in tension to more ductile fractures in compression for unreinforced beams. The study also investigated the effect of lateral surface coverage and reinforcement ratio on the failure mechanism and reinforcement effectiveness.

This paper [21], on the other hand, analysed and compared several reinforcement methods, separately using laminates and sheets and also using a hybrid solution of the two. This paper investigates the use of a non-contact, non-destructive ARAMIS optical system to analyse the static performance of both unreinforced and reinforced laminated veneer lumber (LVL) beams subjected to a four-point bending test. The beams were reinforced with carbon fibre-reinforced polymer (CFRP) sheets and laminates. The strengthening involved bonding CFRP sheets to the outer surfaces in three different configurations, varying in the number of layers and lateral surface coverage. CFRP laminates were also bonded into pre-drilled grooves on the underside of the beams using an epoxy resin adhesive. The study examines strain distribution, stiffness, and ductility using data from the optical measurement system. The strain analysis showed a change from a linear distribution in the compression zone for unreinforced beams to a bilinear distribution for reinforced beams. Stiffness increased by 14% to 45%, depending on whether the CFRP laminates were applied in grooves or the sheets were bonded externally. Similar improvements in ductility were also observed.

As can be seen from the article mentioned above [21], the use of modern testing methods as an optical system makes it possible to obtain results that were previously unavailable. On the other hand, another trend is to look for the application of measuring instruments that are used as a standard in the testing of building materials that are completely different from wood. Three approaches are used to determine the physical-mechanical properties of wood in existing structures: destructive (DT), non-destructive (NDT), and semi-destructive (SDT) testing [22,23,24]. This can be very useful in the work of strengthening existing structures. One such study is described in [25], where the possibilities of testing the mechanical parameters of wood using sclerometry, a standard technique for NDT of concrete, were investigated and evaluated. In this research, the hardness of construction timber, in particular pine, spruce, and fir from Central Europe, was investigated using sclerometric methods. The results of these tests were compared with traditional destructive tests, and correlations were identified. A strong correlation was found between the sclerometric tests and the density of the wood, while the mechanical properties (bending and compressive strength) showed correlations that ranged from strong to moderate. The strength of the correlations varied between different wood species, being strongest for pine and weakest for spruce. The influence of wood defects on the derived correlations was also investigated by considering the knot index. For elements with a small or medium amount of defects, sclerometric methods accurately reflect the physico-mechanical properties. However, the correlations are very weak for wood with a high proportion of defects (knots). This study offers new perspectives on the potential use of semi-destructive methods for the structural evaluation of timber members. It highlights the importance of considering wood species and defect content.

The research problems discussed in the articles included in this Special Issue focused on the effectiveness of the reinforcement of various wooden components or specimens made of solid or glued timber, as determined by experimental tests. Wood is characterised by a complex anatomical structure, which translates into a complex mechanical model, usually defined as orthotropic or transversely isotropic. With the current ability to model the performance of such material models, numerical calculations are increasingly being used to analyse the static performance and evaluate the effectiveness of the reinforcement of timber members.

The laboratory study of wood-CFRP (carbon fibre-reinforced polymer) structural elements, particularly beams, is a topic that many scientists are actively exploring. This research is often complemented by numerical analysis using advanced Finite Element Method (FEM) models. Modern FEM software provides the ability to model various properties and phenomena, such as the orthotropy and plasticity of wood and CFRP, the delamination and mechanical behaviour of adhesive layers, and damage to reinforced elements.

In this regard, the sophistication of the numerical model, the type of material model, and the scope of the calculations performed are fundamental. A summary guideline for FEM modelling of CFRP-wood beams in one of the currently used codes, i.e., Abaqus, is presented in [26].

An analysis of the influence of these issues on the static analysis and the results obtained for bending timber members reinforced with composite laminates is presented in this Special Issue in article [27]. The author of this paper conducts numerical laboratory research on a four-point bending test of a CFRP tape-reinforced glulam beam. The primary objective of this numerical research is to investigate how the complexity of the FEM model affects the calculation results, particularly in terms of stress, deflection, and load-carrying capacity of the glulam beam. In certain scenarios, a simpler model may be sufficient, especially for a structural engineer considering serviceability limit states (allowable deflection of a member) and assuming that the stress should not exceed the yield stress of the wood. In particular, the orthotropic linear elastic model is unsuitable for simulating the mechanical response of CFRP-reinforced glulam because it is only effective for small deflections. Exceptionally, in the case of a cross section divided into laminates, an orthotropic linear elastic model can reproduce reality quite well, but it is limited to small values of the deflection of a glued laminated timber beam. The orthotropic elastic-plastic model agrees well with laboratory tests, so engineers should consider using it when designing CFRP-reinforced glulam beams. The results of FEM calculations are not significantly affected by dividing a section into laminates.
